# Innovations in Clinical Cardiac Electrophysiology: Challenges and Upcoming Solutions in 2018 and Beyond

**DOI:** 10.19102/icrm.2017.081206

**Published:** 2017-12-15

**Authors:** Vaibhav R. Vaidya, Alan Sugure, Samuel J. Asirvatham

**Affiliations:** ^1^Division of Cardiac Electrophysiology, Department of Cardiovascular Diseases, Mayo Clinic, Rochester, MN, USA; ^2^Department of Pediatrics, Mayo Clinic, Rochester, MN, USA

**Keywords:** Arrhythmia, atrial fibrillation, cardiac resynchronization, defibrillation, ablation

Clinical cardiac electrophysiology is a field that continues to develop and innovate at an ever-increasing rate. In the last decade alone, there has been constant innovation and evolution in many aspects of the field, particularly with respect to cardiovascular implantable electronic devices [eg, subcutaneous implantable cardioverter-defibrillator (S-ICD) and the leadless pacemaker], the design of ablation catheters, and the development of novel energy sources for ablation. Despite these advances, however, there still continue to be challenges that remain. In this article, we outline current challenges in our field and innovations to look out for in 2018 and beyond. Due to the explosive growth of device and ablation technology, this review cannot cover all innovative approaches in electrophysiology, but attempts to highlight some of the developments of particular interest.

## Cardiovascular implantable electronic devices

### When can we say goodbye to transvenous pacemakers and defibrillators?

With around one million implants occurring annually worldwide, cardiovascular implantable electronic devices (CIEDs) continue to be heavily utilized in the management of bradycardia, heart failure, and sudden cardiac death. While transvenous lead implantation is the current standard of care, multiple complications can occur, such as device endocarditis, tricuspid valve regurgitation, and venous obstruction. Miniaturized leadless pacemakers that allow single-chamber pacing have been developed, and the Micra^™^ device (Medtronic, Minneapolis, MN, USA) continues to be available commercially.^1–3^ Observational data suggest there have been improvements in long-term outcomes in comparison with historical VVI pacemaker cohorts, but there is no randomized comparison between leadless and conventional transvenous pacing in existence to date. Nevertheless, the next frontier for leadless pacing is developing miniaturized solutions for the gamut of pacing and ICD indications, including dual-chamber pacing, cardiac resynchronization therapy (CRT), and defibrillators with anti-tachycardia pacing capabilities.

***The new and improved S-ICD capable of pacing.*** The S-ICD (Emblem^™^; Boston Scientific, Natick, MA, USA) offers an alternative in circumstances where transvenous leads are best avoided, such as in the young, patients with multiple device infections, and those receiving hemodialysis. The two major limitations of the S-ICD are a lack of pacing for bradycardia and, by extension, an inability to deliver painless anti-tachycardia pacing (ATP) for ventricular tachycardia. Therefore, there is active research into combining S-ICDs with leadless pacing technology. Published reports of both the Micra^™^ (Medtronic, Minneapolis, MN, USA) and NanoStim^™^ (Abbott Laboratories, Chicago, IL, USA) leadless pacemakers implanted concurrently with an S-ICD device demonstrate a lack of crosstalk or double-counting of pacing spikes and native QRS by the S-ICD.^4–6^ Furthermore, there was no dislodgement, malfunction, or reset of the leadless pacemakers following S-ICD-delivered shocks. These studies highlight the feasibility of using a S-ICD and leadless pacemaker concurrently in the future.

A leadless pacemaker capable of unidirectional communication with the S-ICD is currently under development.^7,8^ Acute and chronic animal studies demonstrate a high rate of success in communication between the two devices. There was no interference with S-ICD rhythm discrimination during pacing with the leadless pacemaker. ATP delivery was tested by manual command to the S-ICD and simulated ventricular tachycardia by left ventricular pacing. ATP was delivered in all cases in both the acute and chronic models. First-in-human studies of this cardiac rhythm management device are planned for 2018.

***Selective left ventricular endocardial leadless device—a wise move?*** CRT with placement of a coronary sinus left ventricular lead is widely performed in patients with symptomatic heart failure and evidence of dyssynchrony. The major pitfalls of the current approach, however, include failure to cannulate the coronary sinus, variability in coronary sinus anatomy, lead dislodgement, phrenic nerve pacing, and a lack of response to CRT in one-third of patients. Inadvertent left ventricular endocardial lead placement through a patent foramen ovale or arterial access is usually considered a complication of transvenous device implantation. However, intentional left ventricular endocardial lead placement via a transseptal puncture or the trans-apical route is feasible and results in ventricular remodeling.^9,10^ Multiple studies demonstrate a hemodynamic benefit to endocardial left ventricular pacing in comparison with epicardial pacing.^11,12^ This could be explained by the more physiogenic endocardial to epicardial depolarization and by the existence of the ability to select the pacing site with a deflectable catheter. The obvious drawback of left ventricular endocardial lead placement is the propensity to form thrombus and resultant stroke or systemic embolization. Nevertheless, there exists a subset of patients already on anticoagulation who have not benefitted from CRT either due to technical difficulties in the implantation process or because of a lack of device response; these individuals could be candidates for leadless endocardial left ventricular pacing.

**Figure 1:**
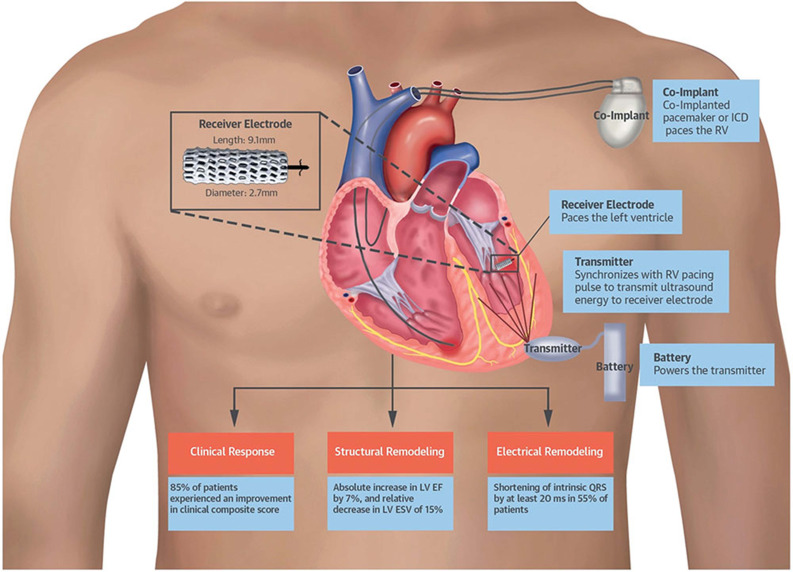
Left ventricular pacing with the WiSE-CRT system (EBR Systems, Sunnyvale, CA, USA). Illustration of device design and function. Used with permission from: Reddy VY, Miller MA, Neuzil P, et al. Cardiac resynchronization therapy with wireless left ventricular endocardial pacing: the SELECT-LV study. *J Am Coll Cardiol.* 2017;69(17):2119–2129.

The WiSE-CRT system (EBR Systems, Sunnyvale, CA, USA) is capable of leadless left ventricular pacing using ultrasound pulses delivered by a subcutaneous generator to a left ventricular endocardial receiver. The endocardial receiver converts the acoustic energy into electric energy for pacing. The WISE-CRT study in 2014 demonstrated the feasibility of this system in 17 patients **(Figure 1)**.^13^ Biventricular pacing was present in 92% of patients at six months, along with an improvement in New York Heart Association functional class and ejection fraction. The trial was suspended early due to multiple cases of pericardial effusion complicating the implant procedure. These complications were attributed to the technical aspects of device delivery rather than to the endocardial lead implant procedure itself, and the SELECT-LV prospective study was performed using a redesigned device delivery system with a distal balloon to facilitate atraumatic entry into the left ventricle. Among 35 patients, the SELECT-LV study again demonstrated the feasibility of this device, with 84.8% of patients demonstrating an improvement in a clinical composite score at six months.^14^ Although pericardial effusions were absent, serious adverse events—including ventricular fibrillation after catheter contact with the left ventricle, embolization of the device to the lower extremity, and femoral artery fistula—were present.

This device has obtained the CE mark in Europe and an investigational device exemption from the United States Food and Drug Administration for further study. A large randomized controlled trial using the WiSE-CRT system is currently underway (NCT02922036).

***Towards percutaneous placement of an epicardial pacemaker and defibrillator***. Potential hurdles to the widespread use of currently designed leadless devices include potential bloodstream infection and an inability to implant in patients who have undergone mechanical tricuspid valve replacement. In addition to overcoming the drawbacks of conventional transvenous devices, epicardial pacing and defibrillation also overcomes these hurdles, but currently requires either sternotomy or a mini-thoracotomy and surgical expertise for implantation. This requires general anesthesia and is more invasive. Recent data suggest surgically placed leads also have a higher rate of failure.^15,16^

A percutaneously implantable epicardial pacemaker and defibrillator system is alluring and would be advantageous in that it would circumvent many of the above problems. Development of a percutaneously implantable epicardial system is currently underway.^17,18^ This system consists of self-expanding, partially insulated leads that are advanced into the pericardium after obtaining pericardial access. Pacing using these leads is feasible, and the partial insulation directs the pacing vector towards the myocardium, avoiding phrenic capture. Defibrillation with this system is feasible, and defibrillation thresholds (DFTs) of as low as 2.5 Joules (J) were obtained. Reduction in DFT is one potential method to avoid defibrillation-related pain, as discussed in the next section. Chronic animal studies and first-in-human studies with these epicardial devices are planned.

### Painless defibrillation—dream or reality?

Although defibrillation is a lifesaving therapy for patients experiencing ventricular tachycardia and/or fibrillation, pain due to shocks results in a reduction of quality of life. Patients with recurrent shocks and electrical storm can suffer tens to even hundreds of shocks, resulting in post-traumatic stress disorder. The ability to painlessly defibrillate the heart would be a game-changer in ICD therapy. The etiology of pain from defibrillation shocks has not been completely elucidated. While the capture of thoracic, abdominal, and other skeletal musculature, and the activation of peripheral nociceptive nerves, are considered the most likely causes of pain, the direct activation of cardiac nerves cannot be ruled out.^19^ In this section, we outline strategies to achieve painless defibrillation that either have been attempted or are on the horizon **(Table 1)**.

***Preventing capture of skeletal muscle and peripheral nerves***. There are two approaches to preventing skeletal muscle and peripheral nerve capture with defibrillation pulses: (1) reduce the DFT to permit lower-energy shocks, or (2) shield the heart from the surrounding tissues.

Sequential defibrillation pulses result in > 50% reduction in DFTs in comparison with those seen with a single shock.^20^ A multistage electrotherapy protocol resulted in atrial mean DFTs as low as 0.16 J in comparison with 1.48 J for routine biphasic defibrillation. The multistage protocol comprised three stages, with stages 1 and 2 using defibrillation shocks and stage 3 incorporating rapid pacing.^21^ Biphasic shock waveforms can be modulated such that defibrillation can be achieved with lower peak voltage of the shock. Boriani et al. designed a plateau-shaped waveform with reduced peak amplitude that resulted in lower pain perception by patients as compared with that associated with conventional tilted waveforms.^22^ Finally, drugs such as sotalol reduce DFTs, but introducing drugs in sufficient concentrations just prior to energy delivery is a challenging task.^23^

Another approach to preventing stimulation of the surrounding tissues is to shield the tissues from the delivery of energy. A Faraday cage operates on the concept that a hollow conductor prevents external charges from acting within it and vice versa, and shields internal charges from the exterior.^24^ This concept was tested with a sock-like elastic defibrillator sleeve positioned over the ventricles in an animal study. Initial studies demonstrated similar DFTs to those of conventional internal defibrillation were achieved, with marked reduction in upper extremity muscle contraction force (1.8 ± 2.0 kg versus 10.6 ± 2.1 kg; p< 0.001).^25^ External defibrillation was not possible despite maximum energy delivery (360 J), due to the shielding effect of the Faraday cage. To overcome this limitation, the investigators developed a series of switches that could disconnect the sock panels as needed to permit external defibrillation. This device again demonstrated reduction in muscle contraction force with internal defibrillation, but was associated with elevated DFTs in comparison with conventional internal defibrillation.^19^

In patients, a thoracotomy would be required to deploy such a sock-electrode. Epicardial partially insulated defibrillation leads can be delivered percutaneously, as discussed previously. These demonstrated successful defibrillation with shocks as low as 2 J, and might result in avoiding capture of the surrounding tissue due to partial insulation.^26^

There are limitations to the concept of complete avoidance of skeletal and peripheral nerve capture. While the threshold for pain with ICD shocks is suggested to be < 0.1 J,^27^ other commonly cited articles provide unconvincing evidence that shocks < 0.1 J are perceived as painless.^28–31^ Current approaches to reducing DFTs, while promising, have not consistently reduced DFTs lower than this 0.1 J threshold.

***Modulating pain perception***. Hunter et al. tested the delivery of “tetanizing” pre-pulses prior to biphasic defibrillation. Their hypothesis was that muscles that are in a fully contracted state before defibrillation cannot be captured by the defibrillation shock.^32^ High-frequency alternating current ramped from an amplitude of 5 V to 100 V was applied for a period of 0.25 to one seconds for muscle capture, and was followed by the biphasic shock. This approach resulted in similar DFTs to those of routine defibrillation. The rate of upper extremity muscle force development was reduced on average by 70%, which is comparable to force development by 0.01 J shocks. While this is promising, defibrillator shock pain can be due to causes other than muscle capture alone. Pain perception could be reduced by delivering sedating or analgesic drug infusions immediately preceding energy delivery, though this has similar practical concerns to the delivery of antiarrhythmics discussed above.

***Nonelectric defibrillation***. An exciting approach to defibrillation would be avoiding the use of electric current altogether. Optogenetics involves the modulation of cellular electrical activity in cells expressing light-sensitive ionic channels. The light-activated cation channel channelrhodopsin-2 (ChR2) channel is activated by light and results in the depolarization of myocardial cells expressing this channel.^33^ In one study, transgenic mice with myocardial ChR2 channel expression were effectively defibrillated with light exposure. Depolarization of a large mass of the myocardium simultaneously resulted in sodium channel inactivation and extinguished fibrillatory wavefronts. Furthermore, an adenoviral vector was used to transfect wild-type mice and resulted in 58% expression of the ChR2 channels. Transfected mice were successfully defibrillated with epicardial illumination, as was a computer model of the human heart.

Another approach to nonelectric defibrillation involves external cardiac cooling. Cardiac cooling results in the reduction of action potential and conduction velocity and the prolongation of the action potential duration. These properties can terminate both atrial and ventricular fibrillation. In a proof-of-concept animal experiment, Peltier elements were used to cool the epicardium (mean: 2.3°C ± 6.5°C).^34^ Cooling resulted in the termination of atrial fibrillation (AF). Furthermore, simulated AF by atrial pacing resulted in noncapture of the myocardium when cooled.

Achieving painless defibrillation would go a long way towards improving the quality of life of ICD patients. Research in this area was once dormant following the failure of painless atrial defibrillation efforts, but is now once again gaining momentum with the approaches outlined above.

## Catheter-based ablation

Catheter-based ablation continues to be highlight efficacious in treating various supraventricular arrhythmias and widely used in the management of AF and ventricular tachycardia. Despite exponential advances, delivering energy to create tissue-specific durable lesions while avoiding collateral damage is a challenge. In this section, we explore upcoming advances that improve our ability to create lesions, map arrhythmias, and incorporate novel targets for ablation.

### Novel ablation modalities on the horizon

***Electroportation: the ablation modality of the future?*** Cardiac ablation has undergone a truly remarkable evolution since first inception for the treatment of patients with refractory supraventricular tachycardia in the mid-19^th^ century. In comparison with prior irreversible treatments such as alcohol injection and surgical transection, cryothermal energy offered the major advantage of the ability to identify the appropriate target area and observe for functional effect following the application of cryothermal energy prior to production of an irreversible lesion. Initially, all approaches were surgical in nature, until a “closed chest”^35,36^ was developed, which is known today as the transvenous approach. Initial practice with the closed chest approach deviated away from the use of cryothermal energy and instead employed direct current (DC) energy^37–39^ and laser.^40^ Results were largely encouraging; however, complications from these approaches, namely barotrauma and perforation, respectively, lead to the study and application of radiofrequency (RF) energy.^41^ The success of RF energy application lead to its use in treating atrial flutter,^42^ AF,^43–45^ and ventricular arrhythmias.^46,47^ To this day, it is the most commonly used energy source.

RF creates ablation lesions through resistive heating of the tissue, with subsequent heat conduction to deeper tissue layers. While RF is largely considered an effective therapy, there has been growing restlessness and concern regarding its significant shortcomings and complications, which are largely the consequence of thermal heat generation. RF has the following major limitations and concerns:

Contact force dependence. Contact force is critical to creating transmural lesions.^48^ With inadequate contact, there is inadequate lesion formation, while excessive contact carries a risk for collateral damage and perforation.^49,50^ The availability of contact force sensing catheters has improved this in some regard; however, more recently, there have been concerns for a significant increase in the risk of atrioesophageal fistulas with these catheters.^51^

Collateral damage. Collateral damage from RF ablation is an ongoing concern. The main areas of particular concern are the esophagus, the coronary artery, the phrenic nerve, and the vagal nerve. Thermal injury from RF can cause ulceration formation or the creation of an atrial-esophageal fistula—a devastating consequence that is often fatal.^51,52^ Due to the proximity of coronary arteries to ablated areas, RF ablation can rarely compromise vascular integrity and function, resulting in coronary spasm, direct vessel trauma, and thromboembolism.^53^ The phrenic nerve (PN) is also another critical structure that is prone to injury in atrial ablations, from RF and cryoablation.^54^ Although 66% of patients achieve complete recovery, others can experience significant morbidity and unwanted symptoms. Additionally, the development of upper gastrointestinal motility complications from injury to the vagus nerve and its branches has been described.^55,56^

Thrombus formation. Coagulation and tissue necrosis-induced thermal energy in RF is associated with a risk of thrombus formation. Although there has been a reduction in procedural thromboembolism events with the use of open-irrigated catheters and heparinization, there is growing evidence for silent cerebral infarcts/lesions and significant concern regarding the long-term consequences of these, particularly dementia and cognitive decline.^57–59^

The recognition of shortcomings and complications from RF, which are fundamentally a consequence of thermal heat generation and collateral damage, has seen a growth and desire to find and test alternate energy approaches. In particular, there has been renewed interest in the application of DC energy for ablation. When DC energy is applied in microsecond pulses, the tissue is exposed to an electric field that results in electroporation. Electroporation is a process in which the cell membrane permeability to ions and molecules is increased upon exposure to electric fields as a consequence of the formation of nanoscale defects or pores.^60^ Depending upon the settings of the DC energy applied (eg, pulse duration, voltage), electroporation is reversible or not, with longer applications promoting longer pore opening and leading to irreversible cell death by apoptosis.

Irreversible electroporation (IRE) is of particular interest to the medical field, with research having established its efficacy in the ablation of solid tumors.^61^ Early results of application of IRE for ablation are encouraging. Animal studies with the use of electroporation have been promising, with applications to pulmonary veins, cardiac muscle, Purkinje fibers, and cardiac ganglia. IRE seems to offer a unique approach that addresses and avoids the limitations of current thermal-based approaches, particularly RF. IRE can be safety and feasibly applied to cardiac tissue, in particular pulmonary vein tissue. Data show that the existence of the ability to create durable transmural lesions^62–67^ without damage to the surrounding structures.^68–72^ More recently, our group has published data on a novel prototype catheter that can assist in performing IRE in the pulmonary veins/atrial tissue.^73^ All of our treated pulmonary veins showed marked electrocardiogram amplitude reduction (61.2% on average) with all lesions transmural and histological analysis showing a loss of cardiomyocytes with preserved structural collagen. Further, there was no significant pulmonary vein stenosis on either computed tomography (CT) scan or histology. Electroporation ablation applied at an energy level to create myocardial lesions has shown sparing of the phrenic nerve.^74^ Further, our group has highlighted that ganglia plexus can be targeted, with such resulting in less atrial myocardial injury in comparison with the application of RF.^75^ Lastly, preliminary data from our group have shown an ability to acutely eradicate Purkinje potentials without major injury to the underlying myocardium.^76^

Irreversible electroporation is a unique therapy capable of overcoming many of the limitations of current thermal-based approaches. While there are still challenges to overcome before it is widely applied in humans (particularly with respect to muscle stimulation), its early promising results places it on the horizon for the future of electrophysiology.

***Intramural needle catheter ablation***. Another drawback of RF ablation is the inability to reliably create transmural lesions, especially when faced with thick myocardium in the region of the interventricular septum or in patients with hypertrophic cardiomyopathy. An intramural needle catheter has been developed to enable the creation of lesions deeper and wider than those of RF ablation.^77,78^ A human feasibility study completed using this catheter in patients with recurrent VT resulted in at least one VT being terminated or noninducible. Complications included two cases of complete heart block and one of cardiac tamponade.^79^ Multiple observational clinical trials utilizing the intramural needle catheters are currently underway (eg, NCT03204981, NCT02799693, NCT01791543) and are expected to shed further light on this novel modality.

***Noninvasive ablation***. Any invasive cardiac procedure carries with it a risk of major complications such as myocardial infarction, stroke, or death. Stereotactic radiation therapy is commonly used for the treatment of solid organ malignancies, and recently there has been considerable interest in utilizing this modality for noninvasive ablation of cardiac tissue in a procedure termed stereotactic arrhythmia radioablation (STAR) by some groups.^80^ Cardiac and respiratory motion is a major challenge in the accurate delivery of radiotherapy, and a fiducial marker such as a pacing catheter placed adjacent to the area of interest can be utilized to guide therapy.^81^

Initial research in swine demonstrated the ability of radiotherapy to form lesions in a variety of locations including the cavotricuspid isthmus, atrioventricular node, and the pulmonary vein-left atrial junction.^82^ Additionally, a study in a patient with recurrent ventricular tachycardia demonstrated the feasibility and safety of stereotactic radiotherapy for cardiac ablation.^81^ Noninvasive ablation with X-ray radiation therapy is also under active research, with preliminary results expected to be released in the next year for some studies is under active research, with preliminary results expected over the next year for some studies (NCT02919618, NCT02661048).

Photon beam therapy is increasingly used for the precise treatment of solid tumors. This modality was investigated in an animal study and showed a dose-dependent reduction in voltage on mapping and the presence of fibrosis and hemorrhage on histology at treated sites, respectively.^83^ In another study, photon beams successfully ablated the atrioventricular node in pigs. However, while the coronary arteries remained intact, there was apoptosis at the sites of myocardial beam entry.^84^ Further research and optimization of stereotactic X-ray radiation and photon beam therapies may usher in an exciting new era of noninvasive ablation.

## Improvements in mapping—seeing is believing

Despite exponential improvements in mapping and ablation technology, the success rates of catheter ablation for certain arrhythmias, such as persistent AF, remain suboptimal.^85^ As compared with that of conditions with high success rates with catheter ablation, such as atrioventricular nodal reentrant tachycardia, our understanding of the mechanism underlying persistent AF is incomplete and continues to evolve. It is expected that as we elucidate the nuances underlying the pathophysiology of persistent AF, mapping and ablation can be better targeted towards these underlying mechanisms.

Two competing theories for the maintenance of AF are the existence or lack thereof of “drivers” or foci of AF that constantly regenerate fibrillatory wavefronts. The lack of existence of such driers is consistent with the multiple-wavelet theory, which posits that wavelets are continuously generated in the fibrillating atrium in a random fashion.^86^ This theory requires a certain critical mass of myocardium to be available for the sustaining of fibrillation, and it is difficult to explain fibrillation in smaller animal using this theory alone.^87,88^ The existence of drivers of AF may also explain reports of persistent AF terminating with point ablation.^89^

**Figure 2:**
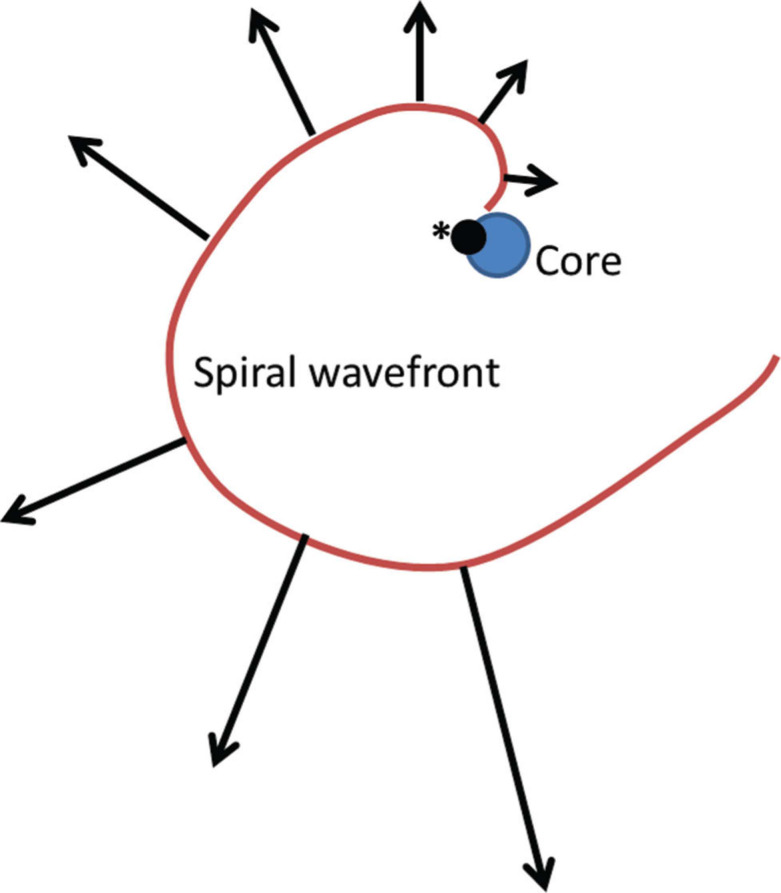
An illustration of a rotor wave. The spiral wavefront appears to spread from an inexcitable core, with a neighboring phase singularity (*). The black arrows denote conduction velocity at various sections of the spiral wavefront. Adapted from Nattel S, Xiong F, Aguilar M. Demystifying rotors and their place in clinical translation of atrial fibrillation mechanisms. *Nat Rev Cardiol.* 2017;14(9):509–520.

Drivers of AF can exist as rapidly discharging foci or, more commonly, in the form of spiral waves or rotors. Spiral waves consist of wavefronts of depolarization emanating around an inexcitable “core” with a low conduction velocity and high curvature close to the core and a greater velocity and lower curvature further away from the core **(Figure 2)**. The existence of spiral waves [or scroll waves in three-dimensional (3D) media] is demonstrated with optical mapping techniques in simulations, animal AF models, and ex-vivo human models.^90,91^ The visualization of rotors is enhanced by using a mathematical technique known as phase analysis, with several excellent publications highlighting the details of this proess.^92,93^ Optical mapping cannot be used in clinical AF cases currently, leading to multiple attempts at visualizing rotors using electrogram mapping, the cornerstone of current mapping approaches.

***Invasive rotor mapping***. Focal impulse and rotor modulation (FIRM) using the RhythmView^™^ system (Topera Medical, Palo Alto, CA, USA) was one of the first commercially available electrogram mapping systems that demonstrated the existence of drivers of AF in the form of rotors and focal sources in humans.^94^ The authors used multielectrode basket catheters to map the atria and proprietary computational algorithms to generate maps, ablating at the site of local rotors or focal impulses. Observational single-center data from the authors demonstrated 2.1 ± 1.0 sources or drovers per patient (70% rotors, 30% focal impulses) that were stable for several minutes. Ablation at these sites resulted in the termination or slowing of AF in 86% of patients. Single-procedure 12-month freedom from AF was excellent in the FIRM-guided group (82.4%) versus in the FIRM-blinded group (44.9%), and the difference between the groups was maintained at three-years post-operation.^95^ A retrospective analysis of the FIRM-blinded group showed a high rate of success if sources were coincidentally eliminated as part of pulmonary vein isolation (PVI).^96^

The initial optimism surrounding FIRM-guided ablation was blunted by the lack of reproducibility of the rotors and the results achieved at other centers, with several of them reporting worse outcomes.^97–99^ A large, randomized trial suggested poor outcomes with FIRM-guided ablation alone but was retracted due to concerns with trial enrollment and registration.^100^ However, other major centers have reported improved outcomes achieved in patients with persistent AF ablation using FIRM-guided mapping,^101,102^ and there are other unfinished trials currently underway using this technology for ablation (NCT02274857, NCT02799043).

***Noninvasive rotor mapping***. Other than the RhythmView^™^ system (Topera Medical, Palo Alto, CA, USA), there are multiple ongoing invasive and noninvasive mapping approaches being considered that are directed towards the localization of rotors and focal impulses in persistent AF. The iECG vest (Medtronic, Minneapolis, MN, USA) includes 252 body surface electrodes and has been used to localize rotors prior to persistent AF ablation.^103^ Unipolar electrograms obtained from the iECG vest (Medtronic, Minneapolis, MN, USA) were converted into phase maps and displayed on 3D biatrial shells constructed from pre-procedure CT scans. Similar to the FIRM study, drivers were present frequently and targeted for ablation in this study. In contrast with the FIRM study, however, the drivers were noted to be nonsustained and to meander frequently, but also to recur in similar regions. The numbers of drivers were noted to increase with increasing AF duration. Driver ablation alone resulted in AF termination in 75% of persistent AF patients, and 85% of these individuals were free of AF at 12 months post-procedure.

Body surface potential mapping utilizes 67 body surface leads to localize atrial areas with the greatest dominant frequency of activation.^104^ The ablation of high-frequency source areas detected on endocardial mapping was noninferior in comparison with PVI (after multiple procedures) and was associated with lower rates of complications.^105^ An observational study is currently underway to validate high-frequency source areas detected by body surface potential mapping using endocardial maps (PERSONALIZE-AF, NCT02497248). Other approaches to mapping rotor are summarized in **Table 2**.

While optical mapping demonstrates rotors in persistent AF patients, the optimal method to map these clinically remains unclear. Catheters with close inter-electrode spacing resulting in improved spatial resolution may enhance our ability to detect rotors with confidence.^106,107^ Advances in mapping rotors and the ablation of these may improve success rates of persistent AF ablation in the future.

### Novel targets for catheter ablation

In this section, we review emerging ablation targets for the treatment of ventricular fibrillation or atrial fibrillation.

***Purkinje fibers***. Synchronous activation of both ventricles is achieved by the rapid spread of depolarization using the Purkinje fibers. In comparison with ventricular myocytes, histologically, Purkinje cells are larger and electrophysiologically have faster action potential upstroke velocity and longer action potential duration.^108^ Purkinje fibers are implicated in a variety of ventricular arrhythmias. Fascicular ventricular tachycardia occurs due to reentrant circuits involving the fascicles and Purkinje fibers. The role of Purkinje fibers in the initiation and maintenance of ventricular fibrillation is increasingly recognized.^109,110^

**Figure 3:**
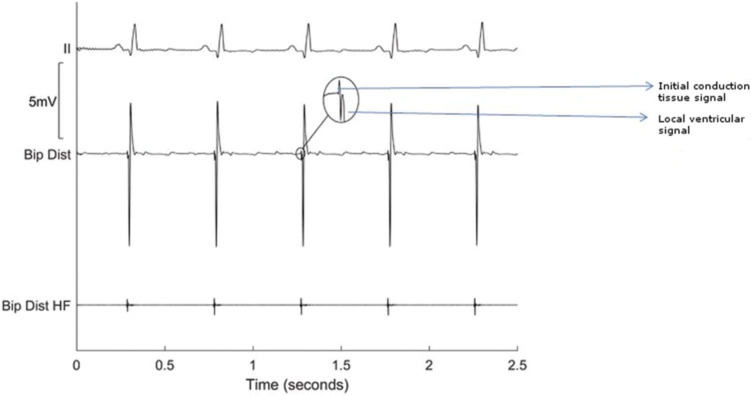
Bipolar distal electrogram from an area with Purkinje fibers. The inset shows a Purkinje signal close to the local myocardial signal. The bipolar distal high-frequency channel demonstrates an electrogram corresponding with the Purkinje signal alone. The high-pass filter is increased to 200 Hz from the conventional level of 30 Hz and proprietary post-processing is performed to reduce noise. Padmanabhan D, Foxall T, Drakulic B, et al. Initial experience with the BioSig PURE EP^™^ signal recording system: an animal laboratory experience. *J Innov Cardiac Rhythm Manage.* 2017;8(4):2690–2699.

Purkinje fibers create distinct high-frequency electrograms due to their fast action potential upstroke. While conventional electrogram processing systems can display Purkinje potentials, it is difficult to visualize these consistently. One reason is because the Purkinje potentials can be buried within the bipolar ventricular electrograms, making it difficult to differentiate between the two. An advanced signal processing system allows for the visualization of the Purkinje signal separate from the local myocardial activation.^111^ This is achieved by using a high-pass filter with additional processing. Local myocardial depolarization can result in large local depolarization signals, and signal processing beyond a linear high-pass filter is required for Purkinje potential display **(Figure 3)**.

***Autonomic nervous system***. The autonomic nervous system regulates several critical physiologic processes, and its role in the genesis of cardiac arrhythmias is increasingly recognized.^112,113^ The paravertebral autonomic ganglia supply sympathetic innervation and the ganglionated plexi (GP) on the extrinsic surface of the heart supply predominantly parasympathetic innervation, although sympathetic components are also present. Modulation of the autonomic nervous system may be a target for the management of multiple disorders that cardiologists face on a regular basis, including AF, ventricular tachycardia, heart failure, and orthostatic hypotension syndromes.

The interplay between the sympathetic and parasympathetic nervous system is implicated in AF. The ablation of the GP has been studied as a treatment for AF. A meta-analysis and subsequent randomized controlled study both demonstrated that GP ablation together with PVI reduced AF recurrence more so than did either strategy alone.^114,115^ The standard lesion set for wide-area circumferential ablation for PVI does very often result in GP ablation and, thus, partial GP ablation is already a part of most AF ablation procedures. Apart from ablation, botulinum toxin can suppress cholinergic output from the GP. In a canine study, botulinum toxin injection into the GP resulted in an increase in pulmonary vein effective refractory periods and AF burden.^116^ Modulation of the autonomic nervous system through novel modalities such as electroporation is under active study and may offer an alternative approach to the management of AF.^75^

Ventricular tachycardia and fibrillation can be suppressed with vagal nerve stimulation in animal models. Vagal nerve stimulation in heart failure has been studied for the endpoints of improvement in cardiac function. The ANTHEM-HF trial showed that vagal nerve stimulation in heart failure patients is feasible and well-tolerated.^117^ However, the NECTAR-HF study failed to demonstrate an improvement in left ventricular end-systolic diameter with vagal stimulation in a randomized trial design.^118^ A much larger randomized study (INNOVATE HF) is currently underway and the ventricular arrhythmia outcomes, if reported, will be of particular interest to electrophysiologists.^119^

Vasovagal syncope is a common disorder faced by internists, cardiologists, and electrophysiologists alike. Treatment options include lifestyle changes, compression stocking use, and drug therapy. However, not all patients respond to treatment, and the condition can be associated with considerable impairment in quality of life. Renal nerve stimulation can increase the mean arterial pressure rapidly in experimental settings.^120,121^ Renal nerve pacing could be a potential treatment in patients with vasodilatory syncope due to vasovagal mechanisms or other disorders affecting the autonomic nervous system in these individuals.

***Cajal cells***. Interstitial cells of Cajal are characterized by elongated cellular processes and mediate pacemaker activity in the gastrointestinal tract. Interstitial Cajal-like cells (also known as telocytes) have been demonstrated in numerous extra-gastrointestinal sites, including in human atria and the ventricles.^122,123^ These cells may interact with stem cells and modulate cardiac regeneration. Although these cells are rare in number in comparison with myocytes, they have multiple elongated processes capable of interfacing with multiple myocytes through gap junctions.^124^ Cajal-like cells possess voltage-gated channels including inward and outward potassium channels.^125^ These cells may be involved in normal impulse propagation within atrial and ventricular myocardium. Interestingly, some authors have noted that these cells disappear from the early post-infarct myocardium, coincident with a period of increased susceptibility to sudden cardiac death.^125^ These cells are present within the myocardium extensions into the pulmonary veins and could play a role in the genesis or maintenance of AF.^126^ As the roles of Cajal-like cells become clearer, these may be candidates for modulation in ventricular fibrillation or atrial arrhythmias.

## Conclusion

Despite the advances in device and ablation technology that have been made, there remain challenges to achieving optimal outcomes in patients with cardiac arrhythmias. Device and ablation technology development continues to explore new frontiers to address these challenges, as described above. The most anticipated developments in cardiac device therapy include multi-site leadless pacing and painless defibrillation, while innovations such as electroporation, rotor mapping, and the identification and use of novel targets for ablation may improve outcomes in persistent AF and ventricular fibrillation.

## Figures and Tables

**Table 1:** Strategies for Painless Defibrillation

Those that Involve the Prevention of Capture of Skeletal Muscle and Peripheral Nerves		Those that Involve the Modulation of Pain Perception	Those that Are Nonelectric Approaches to Defibrillation
Reduction of DFT	Use of methods to shield from defibrillation	Infusion of sedatives/analgesics	Use of epicardial cooling with Peltier elements
Modulation of defibrillation waveform	Use of a Faraday cage	Use of “tetanizing” pulses prior to defibrillation	Optical defibrillation
Employment of sequential low energy shocks	Incorporation of partially insulated epicardial leads		
Drug administration (eg, sotalol)			

**Table 2:** Approaches to Clinical Rotor Mapping

Invasive	
Technique or device	Description
FIRM-guided	See text.
CartoFindert^™^ (Biosense Webster, Diamond Bar, CA, USA)	Basket catheters are used to map atria. Includes proprietary software developed to analyze electrograms and identify rotational activation patterns.^127^
Spatiotemporal dispersion	Areas of electrogram spatiotemporal dispersion are associated with rotors. A study in which the PentaRay^®^ (Biosense Webster, Diamond Bar, CA, USA) was used to detect areas with spatiotemporal dissociation and followed up by ablation resulted in 85% versus 59% arrhythmia-free survival in comparison with conventional ablation at 18 months.^128^
GLOBE^®^ catheter (Kardium Inc., Burnaby, British Columbia, Canada)	A catheter with 16 ribs and 122 flat gold-plated electrodes capable of mapping and ablating, with an inter-electrode distance of 0.8 mm to 1.8 mm. Phase transformation to map rotors is under study.^129^
RMHeartMap 3D (Royal Melbourne Hospital, Parkville, Melbourne, Australia)	Uses 3D atrial maps instead of the two-dimensional maps used by other systems. Atrial shells from electroanatomic maps and electrograms from a basket catheter are analyzed offline to identify demonstrated rotors, especially in areas with high electrode density.^130,131^

Non-invasive	
Technique or device	Description
iECG (Medtronic, Minneapolis, MN, USA)	See text.
Body surface potential mapping	See text.
Noninvasive epi- and endocardial electrophysiology system	Two hundred twenty-four unipolar surface electrodes are used to acquire electrograms that are projected on magnetic resonance imaging atrial shells to detect rotors.^132,133^

FIRM: focal impulse and rotor mapping; 3D: three-dimensional.
